# Recent Experiments Support a Microemulsion Origin of Plasma Membrane Domains: Dependence of Domain Size on Physical Parameters

**DOI:** 10.3390/membranes10080167

**Published:** 2020-07-28

**Authors:** David W. Allender, M. Schick

**Affiliations:** 1 Department of Physics, University of Washington, Seattle, WA 98195, USA; dallende@kent.edu; 2 Department of Physics, Kent State University, Kent, OH 44242, USA

**Keywords:** plasma membrane, rafts, microemulsion, phase-separation, domain size

## Abstract

It is widely, but not universally, believed that the lipids of the plasma membrane are not uniformly distributed, but that “rafts” of sphingolipids and cholesterol float in a “sea” of unsaturated lipids. The physical origin of such heterogeneities is often attributed to a phase coexistence between the two different domains. We argue that this explanation is untenable for several reasons. Further, we note that the results of recent experiments are inconsistent with this picture. However, they are quite consistent with an alternate explanation, namely, that the plasma membrane is a microemulsion of the two kinds of regions. To show this, we briefly review a simplified version of this theory and its phase diagram. We also explicate the dependence of the predicted domain size on four physical parameters. They are the energy cost of gradients in the composition, the spontaneous curvature of the membrane, its bending modulus and its surface tension. Taking values of the latter two from experiment, we obtain domain sizes for several different cell types that vary from 58 to 88 nm.

## 1. Introduction

The “raft” model of the plasma membrane hypothesizes that its lipid constituents are not uniformly mixed, as one would expect from entropic considerations, but are clustered into domains rich in sphingomyelin and cholesterol which float in a “sea” of unsaturated lipids [1,2]. Because the hydrocarbon tails of the sphingomyelin are relatively well-ordered, their domains have a larger areal density than those domains rich in the unsaturated lipids whose acyl chains are more disordered. This difference in areal densities affects the distribution of proteins in the membrane, causing them to favor one form of domain or the other. The proteins cluster and function more efficiently. Thus, physical organization leads to functional organization. There are several reviews of this organizing principle [3,4,5,6].

This hypothesis, however, is not universally accepted. One reason is that there have been no observations of lipid rafts in the plasma membranes of live mammalian cells. In addition, the underlying physical principles which would give rise to such domains, thought to be of nanoscopic size, have not been determined. The explanation most commonly cited is that the rafts and the sea are coexisting phases. This is bolstered by the observation that model membranes, often consisting of a ternary mixture of a high-melting-temperature lipid, such as sphingomyelin, (SM), a low-melting-temperature-lipid, such as dioleoyl-phosphatidylcholine (DOPC), and cholesterol, readily exhibit a separation into two distinct phases [7]. One is rich in the high-melting-temperature lipid and cholesterol. It is denoted "liquid ordered" (lo). The other is rich in the low-melting-temperature lipid, and is denoted "liquid disordered" (ld) [8]. We have argued [9,10,11] that the idea that nanoscopic domains are coexisting liquid phases is an untenable one for several reasons. First it provides no explanation for the nanometer domain size; domains of two coexisting phases are of the size of the system itself, i.e., macroscopic. Second, phase separation has never been observed in the plasma membranes of mammalian cells [12]. It has been observed in giant plasma membrane vesicles (GPMVs) [13], but only at temperatures much lower than physiological ones. To answer this objection, it has been suggested that nanoscopic domains are not the result of an actual phase separation, but of the fluctuations associated with a critical point of a separation that would occur at temperatures below physiological ones [14]. This would require that the cell be self-regulating so that at least two degrees of freedom, such as chemical potential differences of the components, be tightly controlled. An additional argument against this explanation is the following: model membranes whose compositions mimic that of the outer, exoplasmic leaf of the plasma membrane readily exhibit phase separation because of their mix of ordered SM and disordered lipids such as palmitoyloleoyl-phosphatidylcholine (POPC) [7,15]. However, model membranes whose compositions mimic the inner, cytoplasmic leaf show no tendency to phase separate [16]. This is because their lipid composition is almost totally that of disordered, unsaturated, lipids. Hence, even if the coupled leaves of the plasma membrane did phase separate, there would be little composition contrast in the inner leaf. Consequently, the ensuing raft would not be a useful one. It should also be noted that if the domains were in fact coexisting phases, this would have experimentally observable consequences. The heat capacity of the plasma membrane would be infinite, as one could add heat to the system without changing its temperature. Similarly, the compressibility of the plasma membrane would be infinite as one could change its area without changing the surface pressure. These effects are readily observed in more experimentally accessible physically-adsorbed systems [17].

An alternative explanation for the origins of the “rafts” and “sea” is that they result from a microemulsion of regions having liquid-ordered-like and liquid-disordered-like properties [9,10,11]. It is useful to recall that in a bulk system that can exhibit phase separation, its components can be emulsified by the addition of a surfactant, a molecule that essentially drives the surface tension between the phases to zero. A one-phase microemulsion results with two different kinds of regions whose compositions are similar to those of the previously co-existing phases being mixed. The characteristic sizes of these regions is related to the volume fractions of the components. We do not believe that in the plasma membrane the emulsification of lo and ld-like regions is brought about by a molecular analog of a surfactant [18,19]. Rather, the energy penalty of having these different regions in close proximity arises, in part, from the differences between the various spontaneous curvatures of the lipid components and that of the local curvature of the membrane itself. This penalty can be reduced if regions of different membrane curvature are correlated with domains of different spontaneous curvature [20,21]. The domains have a characteristic size that arises from the spontaneous curvatures of the components, and the elastic properties of the plasma membrane, its surface tension, and its bending modulus.

The thermodynamic distinction between the three different suggestions for the origin of rafts is clear. A microemulsion is a well-defined single phase. Its physical properties, such as heat capacity and compressibility, are not singular in general. In addition, like any single phase of a system comprised of *c* different components, it exists in a wide phase space spanned by c+1 degrees of freedom, such as temperature, surface tension, and chemical potentials. In contrast, when two phases, such as lo and ld, are in coexistence, the constraints that the two phases have the same temperature and chemical potentials of all components reduce the number of degrees of freedom of the system from c+1 to *c*. Furthermore, the response functions, such as heat capacity and compressibility of a system of two phases in coexistence, are infinite. Finally, the unconstrained one-phase microemulsion also contrasts with a one-phase system that is constrained to be near a line of critical points. In such a case the number of degrees of freedom is reduced to c−1, which implies that the cell would need to be tightly regulated.

In this paper, we point out that two recent experiments [22,23] are inconsistent with the idea that rafts and sea are regions of two-phase coexistence, but are quite consistent with the idea that they are regions of an emulsion. To do this, we briefly review a simplified theory of the plasma membrane as a microemulsion [9], and its phase diagram [24]. The model employed exhibits both regions of genuine two-phase coexistence as well as a one phase emulsion. We show how the results of the recent experiments are consistent with this phase diagram. In addition, we explicate the manner in which the size of the domains depends on four physical properties, two of them characteristic of the membrane’s lipid composition, and two characteristic of the membrane’s elastic properties. This provides some insight into the manner in which domain size could be controlled.

## 2. Theory

We consider a model plasma membrane that consists of many different lipids. Because the most numerous lipids in the outer leaf are SM, POPC, and cholesterol [25], one should characterize this leaf by at least three order parameters, the local mol fractions of the three species. We have done this elsewhere [11]. Of these three, only two are independent, as the sum of the three local mol fractions is unity. Instead of these two, we shall, for simplicity, restrict ourselves to one order parameter, $\Phi_{o}\left(r\right)$. This measures at point r the difference between the sum of the mol fractions of SM and cholesterol on the one hand, and of POPC on the other. The major components of the inner leaf of the plasma membrane are palmitoyloleoyl-phosphatidylserine, (POPS), palmitoyloleoyl-phosphatidylethanolamine (POPE), POPC, and cholesterol [25]. Consequently, one should describe this leaf by at least three independent order parameters, as in our previous work [11]. Again for simplicity, we describe this leaf by a single order parameter, $\Phi_{i}\left(r\right)$ which represents the local difference between the sum of the mol fractions of POPC and POPS and that of the sum of POPE and cholesterol. This choice was motivated by our recent work [11]. We further simplify by considering only the local average of these order parameters: $\phi \left(r\right)=[\Phi_{o}\left(r\right)+\Phi_{i}\left(r\right)]/2$. Lastly, we assume that the mol fractions of the components are such that the value of ϕ(r) averaged over the surface of the membrane, of area *A*, vanishes.

An expansion of the Helmholtz free energy of the system as a functional of this order parameter contains three terms of interest. The first is the free energy of the flat planar bilayer. It can be written as [24]

(1) $$F_{plane}=\int d^{2}r\left[\frac{a}{2}\phi^{2}\left(r\right)+\frac{b}{2}{\left[\nabla \phi \left(r\right)\right]}^{2}+\frac{c}{4}\phi^{4}\left(r\right)+\frac{g}{2}{\left[\nabla^{2}\phi \left(r\right)\right]}^{2}\right],$$

with *c* and *g* positive. The coefficients *a*, *b*, *c*, and *g* are phenomenological parameters which depend on the subtle interplay of all entropic and energetic contributions to the free energy. If one simply minimizes $F_{plane}$ with respect to the order parameter, then when a>0, the minimum is attained when the order parameter vanishes everywhere. The system is disordered. When a<0, the system undergoes phase separation into two spatially uniform phases characterized by a positive or negative non-vanishing ensemble average of the order parameter. The positive parameter *c* guarantees that the magnitude of the order parameter increases gradually as *a* becomes more negative. Clearly, within this simple minimization, the parameter *a* is proportional to $T-T^{*}\left(\left\{\mu \right\}\right)$, where $T^{*}\left(\left\{\mu \right\}\right)$ is the temperature of phase separation which, in a multi-component system, depends upon the composition, or chemical potential differences, {μ}. The line tension between the coexisting phases is proportional to ${\left(|a|b\right)}^{1/2},$ and vanishes at the transition. We shall refer to the parameter *b* as the gradient energy. The significant length which appears in $F_{planar}$ is the correlation length, which is proportional to ${\left(b/a\right)}^{1/2}$. It is the length over which variations in the composition decay. For example, it is the characteristic width of an interface between coexisting phases. Except near critical points, the correlation length is usually a few nanometers.

The second term of interest is the elastic curvature energy of the membrane. We write it in terms of the local height, h(r), of the membrane above some arbitrary external plane. In the Monge representation, this can be written as [9,26]

(2) $$F_{curv}=\int d^{2}r\frac{1}{2}\left\{\sigma {\left[\nabla h\left(r\right)\right]}^{2}+\kappa {\left[\nabla^{2}h\left(r\right)\right]}^{2}\right\},$$

where σ and κ are the surface tension and bending modulus of the membrane. This term introduces a new length, ${\left(\kappa /\sigma \right)}^{1/2}$, which is characteristic of the membrane. At lengths larger than this, it is the surface tension which is the dominant force that tends to keep the membrane flat, whereas for shorter lengths, it is the bending energy which dominates. In the plasma membrane this length is on the order of 100 nm [27].

Lastly, there is a coupling between the local spontaneous curvature of the membrane, $H_{0}\left(r\right)$, which depends on the composition of both leaves, ϕ(r), and the actual local curvature of the membrane, $\nabla^{2}h\left(r\right)$ [20,21]. In the simplest approximation, $H_{0}\left(r\right)=H_{0}\phi \left(r\right)$, this coupling can be written as [9]

(3) $$F_{coupl}=-\kappa H_{0}\int d^{2}r\nabla^{2}h\left(r\right)\phi \left(r\right).$$

This term introduces a third length, $H_{0}^{-1}$, the membrane’s spontaneous curvature. For the plasma membrane, this is expected to be on the order of tens of nanometers [11]. The total free energy, $F_{tot}$, is simply the sum of the above three terms.

We are most interested in the disordered phase of the system in which the ensemble average of the order parameter vanishes everywhere, <ϕ(r)>=0. The configuration of minimum free energy, $F_{tot}=0$, is that of a flat membrane. The free energy of an arbitrary configuration of the disordered phase is conveniently written in terms of the Fourier components, ϕ(k)

(4) $$\phi \left(k\right)=\frac{1}{A}\int d^{2}r\exp\left(-ik\cdot r\right)\phi \left(r\right),$$

and h(k), of the deviations of the order parameter and the membrane height from their average values. In terms of these Fourier transforms, the total free energy functional of the disordered system can be written, to second order, as

(5) $$\begin{array}{ccc} \frac{F_{tot}\left[\phi ,h\right]}{A} & = & \frac{A}{{\left(2\pi \right)}^{2}}\int d^{2}k\left[\left[\frac{a}{2}+\frac{b}{2}k^{2}+\frac{g}{2}k^{4}\right]\phi \left(k\right)\phi \left(-k\right)\right.\\ & + & \left.\frac{1}{2}\left(\sigma k^{2}+\kappa k^{4}\right)h\left(k\right)h\left(-k\right)-\kappa H_{0}k^{2}h\left(k\right)\phi \left(-k\right)\right] \end{array}$$

The fluctuation free energy of Equation (5) contains three structure factors: <ϕ(k)ϕ(−k)>, <ϕ(k)h(−k)>, and <h(k)h(−k)>. Of these, the one that reveals the most information concerning compositional ordering is <ϕ(k)ϕ(−k)>. The height fluctuations are of less interest, so these degrees of freedom can be integrated over in calculating ensemble averages. The integration can either be carried out explicitly, or the free energy of Equation (5) can simply be minimized with respect to the h(k). Setting the partial derivative of $F_{tot}$ with respect to h(k) to zero and substituting the resulting h(k) into $F_{tot}$, we obtain

(6) $$\begin{array}{ccc} \frac{F_{fluct}\left[\phi \right]}{A} & = & \frac{A}{{\left(2\pi \right)}^{2}}\int d^{2}k\left\{\frac{a}{2}+\frac{b}{2}\left[1-\frac{\kappa^{2}H_{0}^{2}}{b\sigma }\frac{1}{1+(\kappa k^{2}/\sigma )}\right]k^{2}+\frac{g}{2}k^{4}\right\}\phi \left(k\right)\phi \left(-k\right). \end{array}$$

One notes that the effect of coupling the membrane and spontaneous curvatures is to reduce the energy penalty of gradients in the order parameter. Therefore thermal excitations with a wave vector *k* are more easily excited. The wave vector of the mode with the lowest excitation energy in the disordered phase, $k^{*}$, is obtained from $F_{fluct}\left[\phi \right]$ above. Minimizing the excitation energy, we obtain a cubic equation for $k^{2}.$ The solution of this equation is simplified considerably for values such that gσ/bκ<<1, which, as we shall see below, is indeed the case for the plasma membrane. The result is then

(7) $$\begin{array}{ccc} k^{*} & = & 0\;\mathrm{for}\;\kappa H_{0}/{\left(\sigma b\right)}^{1/2}\leq 1,\\ & = & {\left(\frac{\sigma }{\kappa }\right)}^{1/2}{\left[\left(\frac{\kappa H_{0}}{{\left(\sigma b\right)}^{1/2}}\right)-1\right]}^{1/2}\left(1-\frac{g\sigma }{2b\kappa }\frac{\kappa H_{0}}{{\left(\sigma b\right)}^{1/2}}\right)\;\mathrm{for}\;\kappa H_{0}/{\left(\sigma b\right)}^{1/2}\geq 1 \end{array}$$

The implication of this result that the most readily excited modes of the system can have a non-zero wavelength is discussed below.

The value of the gradient energy, *b*, is a few $k_{B}T$ [28], and one expects that *g* is of the order $k_{B}T$nm${}^{2}$. However, the area κ/σ is on the order of $10^{4}$ nm${}^{2}$ for the plasma membrane [29]. Further, the dimensionless coupling $\kappa H_{0}/{\left(\sigma b\right)}^{1/2}$ is less than $10^{2}$, as we shall see below. Hence we ignore the factor proportional to *g* and simply write

(8) $$\begin{array}{ccc} k^{*} & = & 0\;\mathrm{for}\;\kappa H_{0}/{\left(\sigma b\right)}^{1/2}\leq 1,\\ & = & {\left(\frac{\sigma }{\kappa }\right)}^{1/2}{\left[\left(\frac{\kappa H_{0}}{{\left(\sigma b\right)}^{1/2}}\right)-1\right]}^{1/2}\;\mathrm{for}\;\kappa H_{0}/{\left(\sigma b\right)}^{1/2}\geq 1 \end{array}$$

**Figure 1:**
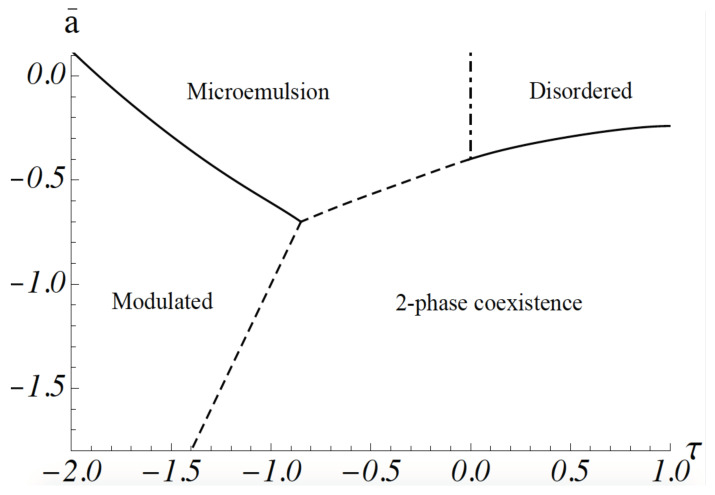
Phase diagram of the model. The dimensionless parameter $\bar{a}=a/c$ is proportional to the difference between the actual temperature and the compositionally-dependent transition temperature. τ is equal to $\left[b/{\left(cg\right)}^{1/2}\right]\left(1-\kappa^{2}H_{0}^{2}/b\sigma \right)$. First-order transitions are shown with dashed lines. The Lifshitz line is shown at τ=0. After reference [24].

The phase diagram of the model can be obtained as follows. As the major effect of the coupling between the fluctuations of the membrane height to those of the composition is to reduce the energy cost of gradients in the composition, the free energy of a configuration of the system is well described by that of Equation (1) alone. However, the coefficient of the gradient energy, *b*, must be replaced by an effective one, $b(1-\kappa^{2}H_{0}^{2}/b\sigma )$. This is a good approximation for the gradient energy except for fluctuations at short distances whose effect on the phase diagram is small. The phase diagram calculated by following this procedure is shown in Figure 1. It was obtained from a molecular dynamics simulation: space is divided into a grid, so that one has a lattice model, but the order-parameter variable at each site is continuous, rather than a discrete one. The equations of motion of the order parameter are the Euler–Lagrange equations obtained from the minimization of the free energy. They are not Newtonian [30]. Such a simulation includes all configurations of the order parameter ϕ(k) [24]. The ordinate is $\bar{a}=a/c$, where *a* and *c* are the coefficients in Equation (1). It is proportional to $T-T^{*}\left(\left\{\mu \right\}\right)$. The abscissa, τ, is equal to $\left[b/{\left(cg\right)}^{1/2}\right]\left(1-\kappa^{2}H_{0}^{2}/b\sigma \right).$ When the dimensionless coupling between concentration and curvature, $\kappa H_{0}/{\left(b\sigma \right)}^{1/2}$ is small, τ is positive. At high temperatures, the system is disordered; that is, the ensemble average of the order parameter, <ϕ(k)> vanishes for all *k*. As the temperature is lowered, a continuous transition, shown by a solid line, occurs to a region of two-phase coexistence: the two phases are characterized by a non-zero value of <ϕ(k=0)>, one positive, one negative. Domains of these phases are macroscopic. At sufficiently stronger values of the coupling, τ<0, the system exhibits a modulated phase of alternating lo-like stripes and ld-like stripes. The ensemble average, <ϕ(k)>≠0 for non-zero wavevector *k*. The parameter *g* of Equation (1) guarantees that the magnitude of <ϕ(k)> increases gradually as the temperature is reduced. At higher temperatures, the system is again disordered in that the ensemble average <ϕ(k)>=0 for all *k*. However, this disordered phase is characterized by structure; this can be observed by scattering experiments that measure the structure factor S(k)∝<ϕ(k)ϕ(−k)>. In the region of sufficiently strong coupling, i.e., $\kappa H_{0}/{\left(b\sigma \right)}^{1/2}>1$, the structure factor has a peak at non-zero *k*, a value which is approximately equal to $k^{*}$. This shows that the disordered system has structure with a domain size that can be taken to be $d\equiv \pi /k^{*}$. As we will see below, this domain size is on the order of tens of nanometers for the plasma membrane. This region of the disordered phase is a microemulsion, an emulsion of lo-like and ld-like domains. There is no phase transition between the microemulsion and the disordered phase which occurs at high temperature and small values of the coupling, and that has a peak in the structure factor at k=0. Thus, the boundary between these disordered phases is arbitrary. It is convenient to take it to be the Lifshitz line, the loci of points at which the peak in the structure factor moves off of the zero wave vector, i.e., where $k^{*}$ of Equation (8) just becomes non-zero. The Lifshitz line is shown in Figure 1 by a dashed-dotted vertical line. There is a line of three-phase coexistence between the region of two-phase coexistence and the microemulsion, shown by a dashed line. There is also a line of three-phase coexistence between the region of two-phase coexistence and the modulated phase. This is also shown by a dashed line.

## 3. Recent Experimental Results

We now consider the results of two recent experiments. In the first, [22] a system of four components, distearoyl-phosphatidylcholine, (DSPC), dipalmitoyloleoyl-phosphatidylcholine, (DOPC), palmitoyloleoyl-phosphatidylcholine, (POPC), and cholesterol was prepared as a GUV in a region of two-phase coexistence. With the addition of cholesterol, the system was brought closer to its critical point and the line tension between the macroscopic phases, was reduced. On the replacement of DOPC by POPC, the system first entered what appears to be a modulated phase. With further replacement, there was another transition to a phase in which no domains could be seen optically. However, from previous experiments [19], this region is known to be characterized by nanodomains. We first observe that having begun in a region of two-phase coexistence and having undergone two phase transitions manifested by morphological transitions, the system exhibiting nanoscopic domains was certainly no longer in a region of two-phase coexistence.

The two observed transitions are, however, quite compatible with our model, and our picture that the region of nanodomains is a microemulsion. The system starts in a region of two-phase coexistence. Hence the compositionally-dependent transition temperature of the system, $T^{*}\left(\left\{\mu \right\}\right)$, is much higher than the actual temperature of the system, *T*. Therefore, the parameter $\bar{a}$, plotted on the ordinate of the phase diagram of Figure 1 is negative and large in magnitude. As DOPC is replaced by POPC, its transition temperature is reduced; hence, $\bar{a}$ increases, becoming less negative. With further replacement of DOPC, $T^{*}$ decreases further and $\bar{a}$ continues to increase. One sees from Figure 1 that there is a large phase space in which paths of increasing $T-T^{*}$ lead to the observed sequence of two-phase coexistence to modulated phase to microemulsion, consistent with experiment. The observation of this sequence of transitions also argues that the system of the GUV is a weakly coupled one; that is, the magnitude of τ in Figure 1 is not large, or equivalently, the dimensionless coupling $\kappa H_{0}/{\left(b\sigma \right)}^{1/2}$ is not large compared to unity.

The other recent experiment of interest [23] detected nanoscopic domains in giant plasma membrane vesicles at temperatures some twenty centigrade degrees higher than the highest temperature at which macroscopic phase separation is observed in them [31]. The absence of macroscopic domains at the higher temperatures clearly indicates that the system with nanodomains does not exhibit two-phase coexistence. Further, nanodomains were observed over a wide range of lipid compositions. This is a strong argument that the nanodomains are in a one-phase region in which one has all of the c+1 degrees of freedom, where *c* is the number of components. It argues against an interpretation that the domains are a result of critical fluctuations as the constraint of being near a critical line eliminates two of those degrees of freedom. Consequently, the cell would have to be tightly controlled. In further support of the emulsion interpretation, one sees from Figure 1 that a transition with increasing temperature from a region of two-phase coexistence, as observed in reference [31], to an emulsion of nanodomains, as observed in reference [23], can certainly be brought about.

## 4. Domain Size and Physical Parameters

In the above theory, the wave vector of the fluctuation in composition that is most easily excited thermally is given by Equation (8). If we arbitrarily choose the size of the domain, *d*, to be given by $d=\pi /k^{*}$, then

(9) $$d=\pi {\left(\frac{\kappa }{\sigma }\right)}^{1/2}{\left[\left(\frac{\kappa H_{0}}{{\left(\sigma b\right)}^{1/2}}\right)-1\right]}^{-1/2}\;\mathrm{for}\;\kappa H_{0}/{\left(\sigma b\right)}^{1/2}\geq 1$$

The domain size depends on four physical parameters: the total spontaneous curvature of the lipid components of the membrane, $H_{0}$; the energy cost of spatial variations in the composition, *b*; and two elastic constants, the membrane’s surface tension, σ, and its bending modulus, κ. These elastic constants were measured several years ago for a variety of cell types [27]. Fourteen values of the surface tensions varied from 1.5 × 10${}^{-5}$ J/m${}^{2}$ to 7.8 × 10${}^{-5}$ J/m${}^{2}$; the bending modulus varies from 1.8 × 10${}^{-19}$ J to 8.7 × 10${}^{-19}$ J. For the gradient energy, we take the reasonable value $b=5k_{B}T=2.1\times 10^{-20}$ J [28]. For the membrane spontaneous curvature, we use $H_{0}$ = 0.05 $\times 10^{-9}$ m${}^{-1}$ as this is comparable to the value calculated recently [11]. That this is indeed reasonable follows from observing that the magnitude of the spontaneous curvature of most lipids varies [32] between 0.01 and $0.4\times 10^{-9}$ m${}^{-1}$. Further, as a lipid on the inner leaf has a spontaneous curvature that is of opposite sign from that of the same lipid on the outer leaf, there is cancellation in the total lipid contribution to the membrane spontaneous curvature. Utilizing these values for the physical parameters, we find that the domain size for these cell types varies from 58 nm to 88 nm, which is in accord with domain sizes estimated from experiments [33,34,35,36]. The same group also measured the elastic properties of GPMVs (denoted PMVs by them) and obtained $\sigma =0.80\times 10^{-5}$ J/m${}^{2}$ and $\kappa =4.1\times 10^{-19}$ J. Presumably the smaller surface tension in the GPMV is due to the lack of a cytoskeleton. In this case one obtains a domain size of 101 nm, somewhat larger than that predicted for the intact cells.

It is also of interest to calculate the dimensionless coupling strength between the membrane configuration and the membrane composition, $\kappa H_{0}/{\left(b\sigma \right)}^{1/2}.$ For the various cell types, we calculate that this coupling varies between 10 and 37. This indicates that the plasma membrane is a strong microemulsion, as opposed to one for which the dimensionless strength is closer to unity. This indication of a strong microemulsion is in accord with somewhat more detailed estimates made earlier [10].

**Figure 2:**
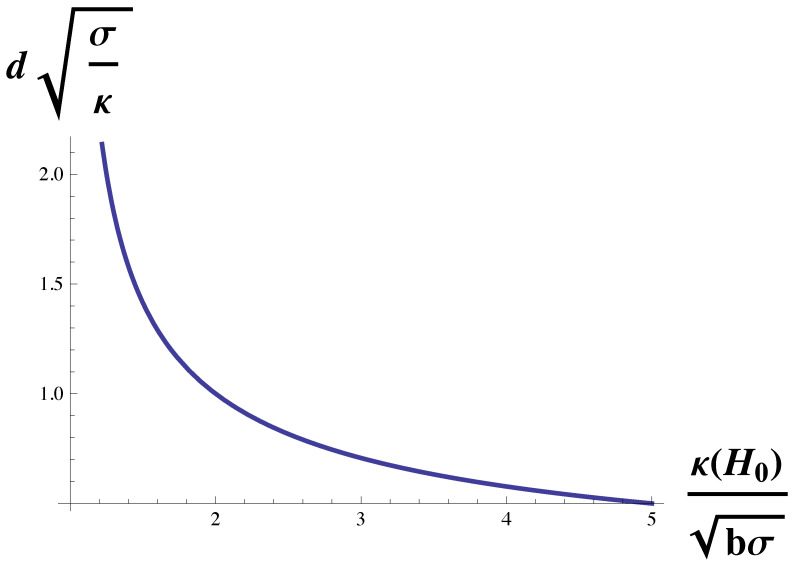
Domain size, *d* in units of ${\left(\kappa /\sigma \right)}^{1/2}$ is plotted vs. $\kappa H_{0}/{\left(b\sigma \right)}^{1/2}$, the spontaneous curvature in units of ${\left(b\sigma \right)}^{1/2}/\kappa$. The domain size diverges at the Lifshitz line, $\kappa H_{0}/{\left(b\sigma \right)}^{1/2}=1.$

The dependence of the domain size on the four parameters of interest, the membrane’s spontaneous curvature, $H_{0}$, its bending modulus, κ, its surface tension, σ, and the gradient energy *b*, can be obtained from Equation (9). We first consider its dependence on the membrane’s spontaneous curvature, $H_{0}$. We rewrite Equation (9) in dimensionless form as

(10) $$d{\left(\frac{\sigma }{\kappa }\right)}^{1/2}=\pi {\left(\frac{1}{\kappa H_{0}/{\left(b\sigma \right)}^{1/2}-1}\right)}^{1/2},$$

which gives the domain size in terms of themembrane’s natural length ${\left(\kappa /\sigma \right)}^{1/2}$. The function on the right-hand side of Equation (10) is plotted in Figure 2. The domain size diverges at the Lifshitz line, $\kappa H_{0}/{\left(b\sigma \right)}^{1/2}=1$ and decreases monotonically with increasing spontaneous curvature.

To isolate the dependence of the domain size on the bending modulus, we multiply Equation (10) by ${\left(\kappa^{2}H_{0}^{2}/b\sigma \right)}^{1/4}$ to obtain

(11) $$d{\left(\frac{\sigma H_{0}^{2}}{b}\right)}^{1/4}=\pi {\left(\frac{\kappa H_{0}/{\left(b\sigma \right)}^{1/2}}{\kappa H_{0}/{\left(b\sigma \right)}^{1/2}-1}\right)}^{1/2}.$$

**Figure 3:**
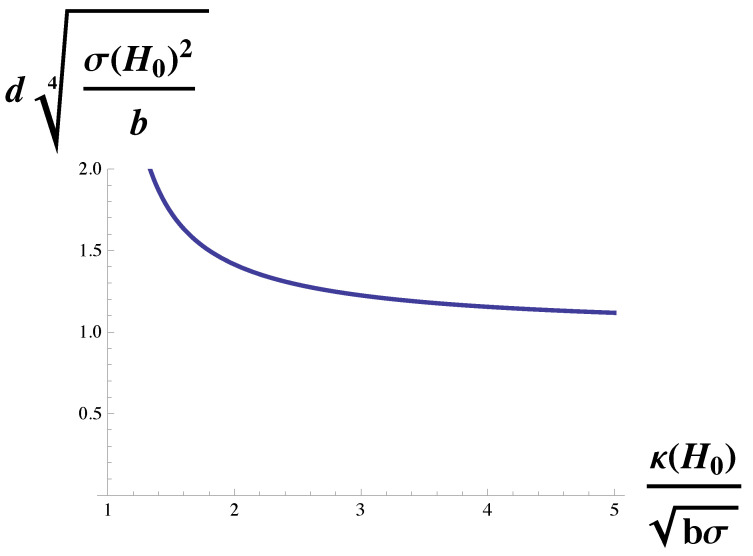
Domain size, *d*, in units of ${\left(b/\sigma H_{0}^{2}\right)}^{1/4}$ is plotted vs. $\kappa H_{0}/{\left(b\sigma \right)}^{1/2}$, the bending modulus in units of ${\left(b\sigma \right)}^{1/2}/H_{0}$. The domain size diverges at the Lifshitz line, $\kappa H_{0}/{\left(b\sigma \right)}^{1/2}=1.$

The function on the right-hand side is plotted in Figure 3. Again, the domain size diverges at the Lifshitz line, $\kappa H_{0}/{\left(b\sigma \right)}^{1/2}=1$. It decreases with increasing bending modulus, but for large modulus, it becomes independent of it and asymptotes to a value of $d\approx \pi {\left(b/\sigma H_{0}^{2}\right)}^{1/4}.$ We noted above that, for the cell types examined in reference [27], the strength of the dimensionless coupling, $\kappa H_{0}/{\left(b\sigma \right)}^{1/2}$ was indeed large compared to unity. It follows that for these cells, the domain size is largely independent of the bending modulus of the plasma membrane, and is given by $d\approx \pi {\left(b/\sigma H_{0}^{2}\right)}^{1/4}$ which, up to a factor of pi, is the geometric mean of two lengths, ${\left(b/\sigma \right)}^{1/2}$ and $H_{0}^{-1}$.

To isolate the dependence of the domain size on the gradient energy, *b*, we simply rewrite Equation (10) in terms of the variable $b\sigma /\kappa^{2}H_{0}^{2}$, linear in *b*, as

(12) $$d{\left(\frac{\sigma }{\kappa }\right)}^{1/2}=\pi {\left(\frac{{\left(b\sigma \right)}^{1/2}/\kappa H_{0}}{1-{\left(b\sigma \right)}^{1/2}/\kappa H_{0}}\right)}^{1/2}.$$

**Figure 4:**
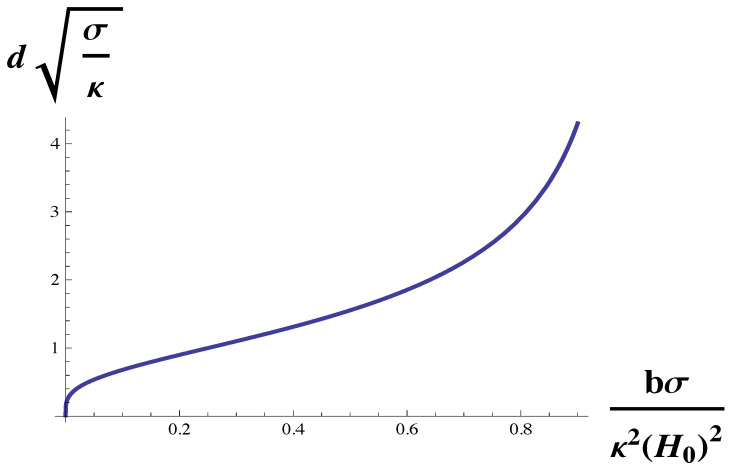
Domain size, *d*, in units of ${\left(\kappa /\sigma \right)}^{1/2}$ is plotted vs. $b\sigma /\kappa^{2}H_{0}^{2}/$, the gradient energy in units of $\kappa^{2}H_{0}^{2}/\sigma .$ The domain size diverges at the Lifshitz line, $b\sigma /\kappa^{2}H_{0}^{2}=1.$

This function is plotted in Figure 4. The domain size vanishes with vanishing gradient energy, and increases with the gradient energy. For sufficiently large gradient energy, the domain size diverges at the Lifshitz line. Recall that in two-phase coexistence, the line tension between phases is proportional to $b^{1/2}$. Thus this dependence of domain size on gradient energy is in agreement with the experimental observation that the size of nanoscopic domains increases with increasing line tension between the macroscopic domains from which they were formed [19].

Lastly, to isolate the dependence of domain size on the membrane surface tension, we multiply Equation (12) by $\kappa H_{0}/{\left(b\sigma \right)}^{1/2}$ to obtain

(13) $$d{\left(\frac{\kappa H_{0}^{2}}{b}\right)}^{1/2}=\pi {\left[\frac{1}{\left[{\left(b\sigma \right)}^{1/2}/\kappa H_{0}\right]\left[1-{\left(b\sigma \right)}^{1/2}/\kappa H_{0}\right]}\right]}^{1/2}.$$

**Figure 5:**
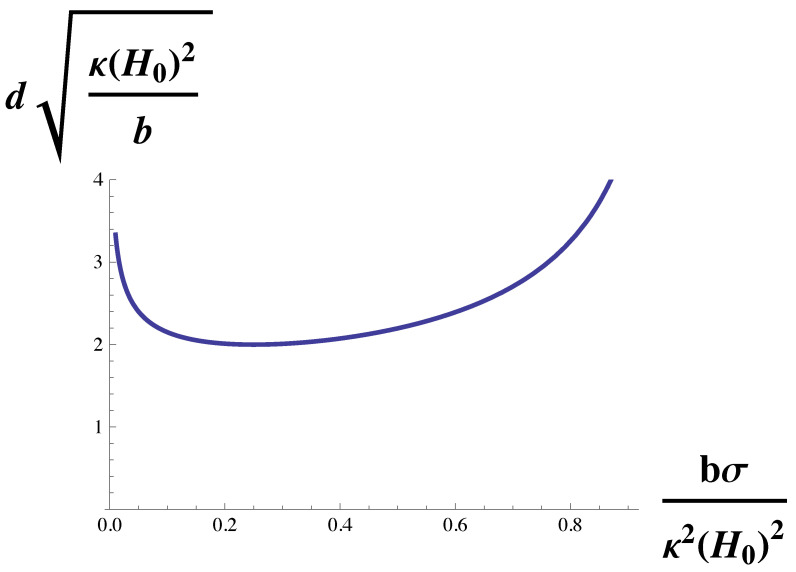
Domain size, *d*, in units of ${\left(b/\kappa H_{0}^{2}\right)}^{1/2}$ is plotted vs. $b\sigma /\kappa^{2}H_{0}^{2}$, the surface tension in units of $\kappa^{2}H_{0}^{2}/b.$ The domain size diverges both as the surface tension goes to zero, and also at the Lifshitz line, $b\sigma /\kappa^{2}H_{0}^{2}=1.$

This dependence on the surface tension, σ, is shown in Figure 5. One sees that the dependence is not monotonic. The domain size diverges at small surface tension, decreases as the tension increases, and then increases with increasing tension as the Lifshitz line is approached. The increase of domain size with tension for large tensions has been experimentally observed both in giant unilamellar vesicles and in GPMVs [37]. We note that this observation, like that of the sequence of transitions observed in reference [22] argues that the GPMVs are also weakly coupled systems, i.e., that $\kappa H_{0}/{\left(b\sigma \right)}^{1/2}$ is not large.

## 5. Discussion

We have noted that the results of two recent experiments are incompatible with the hypothesis that domains in the plasma membrane, or at least in GPMVs and GUVs where they are observed, are regions of distinct lo and ld phases that are in coexistence with one another. The experimental results, however, are well understood in terms of the theory that domains are a microemulsion of lo-like and ld-like regions. The microemulsion is brought about by a coupling of the fluctuations of the membrane’s height and its composition. We noted that experiments indicate that in GPMVs and GUVs, the dimensionless coupling is not a very strong one. This contrasts with the observation we made above: measurements of the plasma membrane’s elastic properties [27] lead us to expect the coupling to be stronger there. The difference would presumably be due to the absence of the cytoskeleton in GPMVs and GUVs. A sufficiently strong coupling leaves open the possibility that in the plasma membrane, rafts might be the result of a modulated phase. This deserves further exploration [38,39].

We have stressed that the picture of phase coexistence implies that the plasma membrane would have singular physical properties, and that the system would have to control tightly at least one, if not two degrees of freedom, such as chemical potential differences. This is in contrast to the microemulsion picture in which the physical properties of the membrane are not singular, and its composition need not be tightly controlled.

We do not imply that the tendency to phase separate is unimportant. If there were not two different regions that tended to separate, there could be no emulsification of those regions. This points to a further shortcoming of the conventional explanation for domains; there is no tendency for the components of the cytoplasmic leaf of the plasma membrane to phase separate due only to their mutual interactions [16]. However, the coupling of membrane height and composition fluctuations tends to separate lipids with different spontaneous curvatures. The cytoplasmic leaf has large mol fractions of lipids whose spontaneous curvatures are large in magnitude, such as POPE, and small in magnitude, such as POPC. Consequently, the emulsion theory predicts that one of the domains in the cytoplasmic leaf will be rich in POPE and cholesterol, while the other will be rich in POPC and POPS [11]. While the conventional explanation can say nothing about the coupling of the domains in the two leaves, the emulsion picture, depending upon the total spontaneous curvature of the two leaves, provides a natural coupling between them [10,11]. It predicts that the domains of SM and cholesterol in the outer leaf will be co-localized with those of POPC and POPS in the inner leaf, as suggested by others [40], and that the domain rich in POPC in the outer leaf will be co-localized with that which is rich in POPE and cholesterol in the inner leaf [11].

Lastly, the coupled fluctuation theory provides a natural origin for the size of the domains. Therefore, it can explicate the dependence of the domain size on several physical parameters: the surface tension and bending modulus of the membrane, and the gradient energy and spontaneous curvatures of the lipids. As we showed, the size of domains predicted by the theory did not vary a great deal over a variety of cell types: from 58 to 88 nm. In particular, we saw that the domain size was relatively independent of the membrane’s bending modulus, as long as the modulus was sufficiently large. Knowledge of the dependence and independence of domains on these physical parameters gives some insight into the manner in which cells can manifest such control over the sizes of rafts, and further, how they might manipulate their properties.
